# Monogenic diabetes: the role of mitochondrial dysfunction and endoplasmic reticulum stress

**DOI:** 10.3389/fcdhc.2025.1659990

**Published:** 2025-11-14

**Authors:** Elif Ozsu

**Affiliations:** Department of Pediatric Endocrinology, Ankara University Medical School, Ankara, Türkiye

**Keywords:** monogenic diabetes, mitochondrial, endoplasmic reticulum stress, children, rare

## Abstract

A diverse range of disorders resulting from various pathophysiological mechanisms are represented by rare forms of diabetes. To date, variants in at least 25 different genes have been identified. Although these forms account for only approximately 6% of all diabetes cases, accurate diagnosis is essential for effective treatment and personalized disease management. Most of these subtypes are monogenic, syndromic, or related to structural abnormalities, providing crucial insights into the genetic and physiological underpinnings of glucose regulation. Clinical clues, such as an early age at onset, the absence of autoantibodies, atypical disease progression, low insulin requirements, and the presence of multi-organ involvement, may indicate a non-classical diabetes phenotype. The classification and recognition of these rare types are clinically significant, especially as advances in genetic technologies continue to expand our understanding of disease mechanisms and therapeutic options. Significantly, the study of rare diabetes forms contributes not only to individualized care but also to the development of novel therapeutic strategies for more common types such as type 1 and type 2 diabetes. The improved understanding of beta-cell function and its genetic regulation through these models has enabled the emergence of precision medicine approaches that extend beyond conventional glycemic control. Mitochondrial diabetes results from mitochondrial defects that impair energy metabolism in pancreatic β-cells, while endoplasmic reticulum (ER) stress—induced by the accumulation of misfolded proteins—triggers β-cell apoptosis. These convergent mechanisms disrupt insulin secretion and glycemic homeostasis, driving diabetes pathogenesis. Elucidating the molecular interplay between mitochondrial dysfunction and ER stress may advance the understanding of disease progression and facilitate the development of targeted therapeutic strategies. This review summarizes the current knowledge on rare forms of diabetes, emphasizing their diagnostic value and therapeutic potential.

## Introduction to monogenic diabetes

Among the rare types of diabetes, monogenic diabetes is a unique and clinically significant subgroup that offers important insights into the disease’s pathophysiology. Individualized care and a precise diagnosis are crucial. About 5% of all cases of diabetes are caused by it. Although severe insulin resistance may occasionally also play a role, the condition is most frequently linked to variations in genes that control beta-cell function and insulin secretion. Insulin therapy, sulfonylureas, or even just dietary control are examples of management techniques. Monogenic diabetes is especially useful for clarifying genotype–phenotype variability and gene–gene interactions, despite its wide range of clinical manifestations. Its clinical significance is highlighted by its early onset, underlying genetic mutations, and potential to affect multiple organ systems.

This group comprises syndromic forms, such as Wolfram and Alström syndromes, as well as monogenic forms, such as neonatal diabetes and maturity-onset diabetes of the young (MODY). Furthermore, diabetes may develop as a side effect of other illnesses, such as autoimmune polyglandular syndromes or cystic fibrosis. These types of diabetes, in contrast to classical type 1 or type 2, are usually distinguished by a strong family history, negative pancreatic autoantibodies, and preserved C-peptide levels—elements that suggest an unusual clinical and biochemical profile. Targeted genetic testing and thorough phenotyping allow for early diagnosis, which optimizes treatment, avoids needless insulin use, and permits suitable genetic counseling. Our knowledge of these uncommon types of diabetes has greatly increased thanks to developments in molecular diagnostics, which have also revealed new pathophysiological mechanisms and the development of medicine approaches.

Pediatric endocrinologists must keep a high index of suspicion when dealing with atypical cases in order to guarantee prompt diagnosis and individualized treatment. Early identification of particular clinical features is made possible by classifying these patients under major subtypes, including neonatal, mitochondrial, endoplasmic reticulum (ER) stress-related, neurological, autoimmune, and severe insulin resistance. Since treatment modalities differ greatly among these subtypes, early detection is essential.

The rare types of diabetes that result from mitochondrial dysfunction and ER stress are the main topic of this review.

## Mitochondrial dysfunction in monogenic diabetes – mitochondrial diabetes

The organelles called mitochondria, sometimes referred to as the cell’s powerhouses, are in charge of generating ATP through the respiratory chain. Only certain mitochondrial components are encoded by the circular genome (mtDNA) found in human mitochondria; nuclear DNA encodes the remaining components. Reduced energy production can result from respiratory chain impairment caused by pathogenic mutations in mtDNA (1–3).

Numerous endocrine abnormalities are linked to specific subtypes of mitochondrial disorders, particularly those involving defects in oxidative phosphorylation. Although the most common endocrine dysfunction is diabetes mellitus (DM), other endocrine glands may also be impacted. Insulin resistance and decreased insulin secretion are the main pathophysiological processes that lead to diabetes (4–6).

## Genetic basis and pathophysiology

Mitochondrial DNA (mtDNA) is maternally inherited because paternal mitochondria are not retained during fertilization. Due to its low recombination and high variant rates, variants can accumulate over time. These mutations may lead to either a homoplasmic or heteroplasmic state, depending on whether mutant or wild-type (WT) mtDNA predominates. Most pathogenic mtDNA mutations remain heteroplasmic, as homoplasmic presence often impairs cell viability. Mitochondrial fusion may allow functional complementation, where WT mtDNA compensates for mutant variants (7, 8).

Mitochondrial disorders are estimated to affect 1 in 5000 individuals. These disorders may result from genetic defects in mtDNA—either de novo mutations or those inherited maternally—or from mutations in nuclear DNA (often autosomal recessive) encoding structural and functional mitochondrial proteins. Due to their heterogeneity, variable clinical manifestations, non–age–specific onset, and multi-organ involvement, mitochondrial diseases often result in diagnostic delays. Pathogenic variants in more than 300 nuclear genes cause mitochondrial disease, some of which are associated with endocrine abnormalities (9).

In addition to diabetes as a typical initial finding, endocrine dysfunctions such as hypogonadism, hypoparathyroidism, and adrenal insufficiency may also occur. The clinical spectrum is broad and may include muscle weakness, sensorineural hearing loss, and multisystem involvement affecting the heart, liver, and central nervous system (10–12).

Endocrine organs with high energy demands are frequently affected, and complications may develop. Mitochondrial endocrinopathies are typically characterized by hormone deficiencies due to the energy dependence of hormone synthesis and secretion. Although all endocrine tissues may theoretically be affected, certain organs appear to be more vulnerable. Notably, diabetes mellitus is present in 11–15% of patients. The average age of diagnosis is in the third decade, but early-onset cases have also been documented. The prevalence of mitochondrial diabetes has been shown to increase with age (10).

## The m.3243A>G variant and clinical spectrum

Pathogenic point mutations have been identified in many regions of the mitochondrial genome, with a notable concentration in tRNA genes. Among the mtDNA variants causing diabetes, the most common is the A3243G mutation in the tRNA Leu (UUR) gene. In a patient presenting with both diabetes and bilateral sensorineural hearing loss, there is a high likelihood of the m.3243A>G variant or another pathogenic mitochondrial mutation, and thus, whole mitochondrial genome sequencing should be considered (13).

In Northwestern Europe, diabetes and hearing loss are the most prominent clinical features. However, the reasons for phenotypic variability remain unclear. Some evidence suggests that higher heteroplasmy levels in the brain may predispose to MELAS (Epilepsy Associated With Mitochondrial Encephalomyopathy, Lactic Acidosis, and Stroke-Like Episodes), while higher levels in the pancreas may predispose to diabetes. Heteroplasmy levels in blood are unreliable predictors of clinical phenotype. Variability in expression may be related to genetic background or patient selection criteria (14).

The m.3243A>G mutation, initially discovered in individuals with MELAS (Mitochondrial Encephalomyopathy, Lactic Acidosis, and Stroke-like episodes), is classically linked to severe multisystem involvement and early mortality. Sensorineural hearing loss—most prominently at frequencies above 5 kHz—often precedes the onset of diabetes in affected individuals. However, unlike classical MELAS many carriers of this mutation do not exhibit overt neurological deficits. In a Dutch cohort study involving 100 individuals with diabetes who harbored the m.3243A>G variant, only one patient demonstrated clinical features consistent with MELAS, suggesting that diabetes may be the predominant manifestation in certain populations. Conversely, in Japan, the mutation is more commonly associated with MELAS. Additionally, some cases have documented progressive renal dysfunction resembling Alport syndrome in association with this mutation. Supporting this, a recent French investigation reported renal involvement in approximately 28% of carriers (14).

Certain mtDNA deletion/duplication rearrangements are primarily associated with diabetes. Ballinger et al. reported a maternally inherited family case of hearing loss and insulin-dependent diabetes due to a heteroplasmic partial duplication with a 10.4 kb mtDNA deletion. In this family, diabetes was the predominant phenotype, whereas in syndromes like Pearson, Kearns–Sayre, and progressive external ophthalmoplegia (PEO), diabetes is more often a secondary feature. Studies in Pearson syndrome reveal marked heteroplasmy variability across fibroblasts, which may also be observed in other tissues and mutations. Some of the identified patients had insulin-dependent diabetes, while others had non–insulin-dependent diabetes. Following these findings, it became evident that the diabetes phenotype associated with mitochondrial dysfunction is variable, ranging from insulin-dependent to non–insulin–dependent (15).

## Mechanisms of β-cell failure and insulin resistance

Mitochondrial diabetes can result from either impaired insulin secretion or reduced insulin sensitivity. In primary mitochondrial defects, reduced oxidative phosphorylation capacity can lead to increased ROS (reactive oxygen radicals), which contributes to beta-cell dysfunction and insufficient insulin release (15, 16).

Even before beta-cell dysfunction is evident, insulin resistance in skeletal muscle has been observed in mitochondrial diabetes. The detailed pathophysiology of muscle insulin resistance in mitochondrial diseases remains under investigation. Increased oxidative stress due to reduced mitochondrial oxidative phosphorylation capacity may underlie tissue-specific insulin resistance. Interestingly, in certain contexts, enhanced mitochondrial respiration may offer protection against diabetes, as demonstrated in animal models of ANT1 deficiency, which impairs ATP transport (15).

MIDD (Maternally Inherited Diabetes and Deafness) is associated with a high prevalence of hearing loss, affecting up to 90% of individuals. Factors like reduced physical activity and hyperglycemia exacerbate insulin sensitivity issues. These complexities underline the need for comprehensive diagnostic and management strategies to avoid misdiagnosis, especially between type 1 and type 2 diabetes. Some patients may also present with abnormal autoimmune markers, which are unusual in MIDD but may indicate secondary responses linked to mitochondrial dysfunction impacting beta-cell function (17–19).In mitochondrial diabetes, hearing loss represents the most prominent extra-clinical feature aiding in patient identification. In MIDD, hearing loss typically precedes diabetes diagnosis by an average of six years, highlighting the syndrome’s progressive and multisystemic nature. Animal models suggest that diabetes arises from impaired glucose-stimulated insulin secretion and a reduction in beta-cell mass (15).

Ultimately, impaired pancreatic beta-cell function underlies abnormal glucose tolerance in MIDD. Secondary contributors like hyperglycemia and reduced physical activity further decrease insulin sensitivity (2, 3). These findings emphasize the need for a comprehensive diagnostic and therapeutic approach to endocrine dysfunction in mitochondrial disorders (15).

Diabetes may be the first manifestation of mitochondrial disease. In Wolfram syndrome (WS), patients often present initially with non-autoimmune insulin-deficient diabetes (typically diagnosed around age six), followed by optic atrophy, leading to WS diagnosis. In maternally inherited diabetes and deafness (MIDD), hearing loss often precedes diabetes but may also follow it. Approximately 1% of individuals with MIDD are initially misdiagnosed with type 1 or type 2 diabetes. Diabetes as the initial manifestation has even been reported in Friedreich’s ataxia (FA), which primarily presents with neurological symptoms (15).

Although uncommon, diabetic ketoacidosis (DKA) has been reported in mitochondrial diseases such as FA, MELAS, Kearns–Sayre syndrome (KSS), and WS. Hyperosmolar hyperglycemia has also been documented in KSS. The clinical course of patients can vary significantly, even within the same family (15).

## Diagnosis and molecular testing

Age at diagnosis is not a definitive criterion for distinguishing mitochondrial diabetes from other forms of monogenic diabetes, as patients are often diagnosed in their 30s. This may lead to confusion with diagnoses such as MODY or LADA (Latent autoimmune diabetes in adults) (15).

In mitochondrial diabetes, an elevation in autoimmune markers is not generally anticipated; nevertheless, there are rare cases in which these markers have been detected as positive. In MIDD, the presence of low-grade ICA positivity in the absence of GAD (Anti-glutamic acid decarboxylase) antibodies has been suggested to reflect a secondary autoimmune response triggered by partial beta-cell damage resulting from mitochondrial dysfunction (15).

Genetic approaches to diagnosing mitochondrial diseases vary across countries and laboratories. Traditional methods, such as pyrosequencing, remain in use for common variants like m.3243A>G, but many laboratories now employ direct mtDNA amplification and next-generation sequencing (NGS). NGS provides high coverage depth for sensitive detection and quantification of mutant loads. However, blood-only testing may be insufficient due to tissue-specific variant expression and the decrease in heteroplasmy over time. Thus, non-invasive samples, such as urinary sediment or a muscle biopsy, may be required (9).

More than 300 nuclear genes are known to be associated with mitochondrial disease, though few are directly related to endocrine dysfunction. Whole-exome sequencing (WES) and whole-genome sequencing (WGS) enhance diagnostic yield and allow detection of mtDNA variants as well. WGS is superior for identifying non-coding variants and copy number alterations. Nevertheless, analysis strategies and gene selection vary depending on the application (20).

## Clinical management and treatment

Due to the high risk of insulin deficiency in mitochondrial diabetes, early assessment of insulin secretory reserve via C-peptide testing is recommended, along with patient education on home ketone testing. Metformin should be avoided due to the risk of lactic acidosis. Sulfonylureas may be effective, but they should be initiated at low doses with close glucose monitoring due to the risk of hypoglycemia. Gastrointestinal dysfunction often leads to nutritional challenges. Among available therapies, DPP-4 inhibitors are preferred over GLP-1 receptor agonists based on small case series. Although SGLT2 inhibitors may offer cardio- and renoprotective effects in mitochondrial diabetes, the associated risk of ketoacidosis necessitates patient education on ketone monitoring and timely drug discontinuation (20).

Mitochondrial diabetes (MD) requires a tailored diagnostic and therapeutic approach distinct from conventional strategies. These patients often exhibit insulin secretory defects and altered ketone metabolism. The Newcastle guidelines recommend insulin initiation in the presence of ketonuria or low C-peptide. Mitochondrial respiratory chain defects increase the intracellular NADH/NAD^+^ ratio, shifting ketone production toward β-hydroxybutyrate over acetoacetate. Thus, serum β-hydroxybutyrate measurement is more reliable than urinary acetoacetate in suspected ketoacidosis (20).

Although insulin therapy is commonly reported in MD case series, evidence on the efficacy and safety of other antidiabetic agents remains limited. Mitochondrial diabetes does not uniformly require insulin therapy, as not all cases are insulin-dependent. There are no controlled trials assessing oral agents in this population. Currently, only insulin, metformin, and liraglutide are approved for individuals under 18. Treatment plans must be individualized based on risk–benefit assessment (20).

Effective management of MIDD involves genetic counseling, monitoring mitochondrial function, and personalized therapies, with a focus on lifestyle modifications to improve insulin sensitivity. It is essential to differentiate MIDD from other forms of diabetes, such as type 1 and type 2 diabetes, to avoid misdiagnosis (21, 22).

Although lifestyle modifications are advised for people with mitochondrial diabetes, their implementation can be challenging because of the supporting data and the existence of comorbid conditions like cognitive dysfunction, low bone density, cardiomyopathy, gastrointestinal disorders, and mobility issues.

In conclusion, since mitochondrial diabetes mellitus is a possible subtype of monogenic diabetes, clinicians should be on the lookout for it. This condition can manifest as progressive β-cell dysfunction that is maternally inherited and sensorineural hearing loss. It is recommended to screen for the m.3243A>G mutation in diabetic patients under 50 years of age, with a BMI below 30, who are autoantibody-negative and exhibit multisystem involvement. In cases where this mutation is absent but significant neurological manifestations are present, comprehensive mtDNA analysis should be considered. **Table 1** summarizes the types of mitochondrial diabetes.

**Table 1:** Genetic disorders associated with mitochondrial DNA and nuclear DNA variants.

Category	Genetic basis	Clinical syndrome/example	Diabetes features	Associated manifestations	Therapeutic approach
mtDNA point mutations	- Most common: m.3243A>G (*MT-TL1*)- Others: m.14709T>C, m.12258C>A	- Maternally Inherited Diabetes and Deafness (MIDD)- MELAS- MERRF	- Young adult onset- Predominant insulin secretory defect- Initially responsive to oral agents, but progressive insulin requirement	- Sensorineural hearing loss- Myopathy- Stroke-like episodes	- Metformin generally contraindicated (risk of lactic acidosis)- Limited efficacy of sulfonylureas- Insulin therapy usually required- SGLT2 inhibitors should be used with caution
Large-scale mtDNA deletions	Recurrent or large-scale deletions	- Kearns–Sayre syndrome (KSS)- Pearson syndrome	- Early onset- Rapidly progressive β-cell failure- Insulin therapy typically required	- Retinitis pigmentosa- Cardiac conduction defects- Exocrine pancreatic insufficiency- Sideroblastic anemia	- Early insulin therapy is usually necessary- Multidisciplinary management for cardiac and hematologic complications
Nuclear DNA mutations	*POLG, TWNK, OPA1, LRPPRC* and others	- POLG-related disorders- Leigh-like syndromes	- Heterogeneous phenotype- β-cell dysfunction with multisystem involvement	- Neurological impairment- Myopathy- Growth retardation- Hepatic dysfunction	- Early insulin requirement in most cases- Mitochondrial cofactors (e.g., coenzyme Q10, riboflavin, L-carnitine) may provide supportive benefit- Multidisciplinary follow-up required

POLG (DNA polymerase gamma) TWNK (Twinkle helicase) OPA1 (Optic atrophy 1) LRPPRC (Leucine-rich pentatricopeptide repeat-containing protein

## Endoplasmic reticulum and endoplasmic reticulum stress in endocrine disorders

One important organelle that is involved in many vital cellular functions, such as protein folding, calcium storage, lipid metabolism, autophagy, and apoptosis regulation, is the endoplasmic reticulum (ER). Specialized chaperone proteins aid in the precise folding of newly synthesized proteins inside the ER. Only correctly folded proteins make it to the Golgi apparatus, where they work as secreted or membrane-bound proteins. Conversely, misfolded proteins are kept in the ER and removed by the endoplasmic reticulum-associated degradation (ERAD) pathway (23).

The endoplasmic reticulum uses its chaperone system and ER-associated degradation (ERAD) pathways to preserve protein homeostasis under physiological conditions. However, misfolded proteins can build up and overwhelm the ER’s capacity, causing cytotoxicity, when protein folding demand rises—due to cellular stress or genetic mutations. Eukaryotic cells use a protective signaling network called the unfolded protein response (UPR) to regain homeostasis. However, apoptosis—a mechanism increasingly implicated in the development of a number of human diseases—can result from prolonged or severe ER stress. Because of their limited ability to regenerate, neurons are especially susceptible to ER stress-mediated cell death, which has been linked to neurodegenerative diseases like Parkinson’s disease, Alzheimer’s disease, and polyglutamine expansion disorders (24).

In mammals, the UPR operates through three well-characterized pathways: PKR-like ER kinase (PERK), activating transcription factor 6 (ATF6), and inositol-requiring enzyme 1 (IRE1). These pathways function coordinately to suppress general protein translation and enhance the expression of UPR chaperones and ERAD components.

## Endoplasmic reticulum stress in monogenic diabetes

Loss of endoplasmic reticulum (ER) homeostasis plays a critical role in β-cell dysfunction and apoptosis, contributing to the development of both type 1 and type 2 diabetes. As a result, the ER has become a key area of focus in diabetes research. In this context, our approach centers on elucidating how ER stress leads to β-cell failure, particularly through the study of monogenic diabetes forms linked to ER dysfunction. Gaining insights into these rare genetic conditions—such as Wolfram syndrome, where ER stress is a major driver of disease—may support the development of targeted, individualized therapies. This section explores the clinical features and molecular mechanisms underlying ER stress–related monogenic diabetes (25).

## Genetic basis and pathophysiology

Pancreatic β-cells are highly dependent on the functional integrity of the endoplasmic reticulum (ER) to support efficient insulin production and secretion. Disruption of ER homeostasis—caused by factors such as genetic alterations, infections, or chronic hyperglycemia—leads to the accumulation of unfolded or misfolded proteins. This activates the unfolded protein response (UPR), a cellular mechanism designed to restore ER function. However, when ER stress is sustained or excessive, the protective capacity of the UPR becomes insufficient, resulting in β-cell death through apoptosis (26).

ER stress is implicated in both type 1 diabetes (via autoimmune-mediated β-cell destruction) and type 2 diabetes (through β-cell exhaustion and insulin resistance). In monogenic disorders like Wolfram syndrome, mutations in WFS1 cause unresolved ER stress and direct β-cell damage. Experimental models, including PERK-deficient mice, demonstrate that failure of key ER stress sensors leads to β-cell loss and early-onset diabetes, underscoring the pathogenic importance of ER dysfunction in diabetes (26).

Pancreatic β-cells are required to adjust to fluctuating insulin demands, particularly during periods of increased secretory activity. While the unfolded protein response (UPR) is not directly responsible for insulin release, it plays a vital role in the proper folding and processing of proinsulin within the endoplasmic reticulum (ER). Mature insulin is stored in secretory granules and released in response to circulating glucose concentrations. Under physiological conditions, proinsulin production supports β-cell proliferation but simultaneously imposes a burden on the ER. In pathological contexts—such as insulin resistance or the presence of misfolded mutant proinsulin—this burden surpasses the adaptive capacity of the UPR, ultimately resulting in loss of β-cell mass and impaired function (27, 28).

Several murine models have been instrumental in elucidating the role of ER stress in diabetes pathogenesis. The Akita mouse model, for example, harbors a heterozygous missense mutation (C96Y) in the Ins2 gene, impairing disulfide bond formation and causing proinsulin misfolding. This leads to persistent ER stress and chronic activation of UPR branches such as ATF6 and IRE1-XBP1. Prior to the onset of hyperglycemia, C/EBP transcription factor accumulates in pancreatic islets, downregulating ATF6 activity and BiP expression, thereby promoting apoptosis mediated by unresolved ER stress. In humans, mutations in the INS gene—including the C96Y variant—have been associated with neonatal diabetes, and the Akita mouse is widely regarded as a relevant model for MODY-like monogenic diabetes (27, 28).

## ER stress–related diabetes

Wolfram syndrome is a neurodegenerative disorder caused by ER dysfunction due to pathogenic WFS1 variants. Misfolded proteins trigger ER stress and mitochondrial impairment, leading to β-cell and neuronal degeneration. Clinical severity varies; milder forms often involve missense variants, such as p.R558C. Wolfram type 2 (WFS2), linked to CISD2 mutations, is characterized by CDGSH iron sulfur domain protein 2, which shares core features but exhibits unique signs, including gastrointestinal ulceration. CISD2 encodes a protein at the ER and mitochondrial membrane, involved in apoptosis and autophagy regulation (29).

In Wolfram syndrome, diabetes mellitus typically emerges first, often around the age of six, followed by the onset of optic atrophy by approximately eleven years of age. Over the subsequent decades, additional features such as diabetes insipidus, sensorineural hearing loss, neurogenic bladder, and obstructive sleep apnea gradually appeared. In advanced stages, patients may develop dysphagia, ataxia, and central sleep apnea, commonly attributed to progressive cerebellar and brainstem atrophy (30).

The clinical spectrum of the disorder is heterogeneous, with disease severity often influenced by the underlying genetic variants. Missense mutations and compound heterozygous genotypes tend to correlate with milder phenotypes. One such example is the WFS1 p.R558C variant, observed in roughly 3% of the Ashkenazi Jewish population, which has been associated with a less severe clinical presentation (30).

A genetically and clinically distinct form, Wolfram syndrome type 2 (WFS2), is caused by pathogenic variants in the CISD2 gene. The CISD2-encoded protein localizes to both the endoplasmic reticulum and the outer mitochondrial membrane and plays roles in apoptosis regulation and autophagy. While WFS2 shares core features such as diabetes mellitus and optic atrophy with WFS1-related disease, it is uniquely characterized by upper gastrointestinal ulceration and bleeding, offering a key clinical differentiator (30).

Variants in EIF2AK3, the gene encoding PERK, cause Wolcott–Rallison syndrome, a condition characterized by permanent neonatal or early-infantile diabetes. This and other findings have directed substantial research attention to the link between ER stress and diabetes. Among consanguineous populations, Wolcott–Rallison syndrome stands out as the predominant monogenic cause of permanent neonatal diabetes. However, a case has also been reported in which diabetes mellitus was diagnosed at the age of 14 (31).

In conclusion, the endoplasmic reticulum (ER) is essential for insulin biosynthesis within pancreatic β-cells through its role in proper protein folding. ER function can be disrupted by various stressors, leading to the accumulation of misfolded proteins and activation of the unfolded protein response (UPR). While the UPR initially acts as a compensatory mechanism to restore ER homeostasis, prolonged or unresolved ER stress ultimately triggers β-cell apoptosis and loss of functional β-cell mass. This mechanism has been implicated not only in type 1 and type 2 diabetes but also in monogenic and syndromic forms such as Wolfram syndrome. The consistent involvement of ER stress across multiple diabetes subtypes highlights its central role in disease pathogenesis and progression. Therefore, therapeutic strategies aimed at alleviating ER stress or enhancing adaptive UPR signaling represent a promising avenue for preserving β-cell integrity and improving metabolic outcomes in patients with both rare and common forms of diabetes.

## Future directions in research

Accurate diagnosis and effective treatment of patients with monogenic and syndromic forms of diabetes are significantly challenged by clinical and genetic heterogeneity as well as variable expressivity. Genetic testing is the most effective tool available to physicians for diagnosing genetic forms of diabetes, offering genetic education and counseling, and improving disease management. The American Diabetes Association recommends genetic testing for all children diagnosed with diabetes within the first six months of life, as well as for individuals with diabetes that does not resemble type 1 or type 2 diabetes and is present in successive generations during early adulthood.

In addition, cases of insulin-deficient, autoantibody-negative diabetes in childhood and early adulthood—particularly when accompanied by neurological dysfunction or other systemic abnormalities—should also be investigated for underlying genetic causes.

Genetic testing based on next-generation sequencing (NGS) technologies, including exome and whole-genome sequencing, can play a key role in identifying DNA variants highly associated with each patient’s clinical signs and symptoms. The routine use of NGS-based testing will not only assist clinicians, medical geneticists, and genetic counselors in providing personalized guidance but will also serve as the first step toward developing tailored therapeutic strategies for individuals with monogenic and syndromic diabetes (**Table 2**).

**Table 2:** Diabetes types associated with ER stress.

Gene	Protein	Syndrome/Disease	Inheritance
INS	Insulin	Permanent Neonatal Diabetes Mellitus	Autosomal Dominant
EIF2AK3	PERK	Wolcott–Rallison Syndrome	Autosomal Recessive
EIF2S3	eIF2γ	MEHMO Syndrome	X-linked
PPP1R15B	CReP	MSSGM2 Syndrome	Autosomal Recessive
DNAJC3	p58IPK	ACPHD (Pancreatic and Neurologic Defects)	Autosomal Recessive
IER3IP1	IER3IP1	MEDS (Microcephaly, Epilepsy, and Diabetes)	Autosomal Recessive
WFS1	Wolframin	Wolfram Syndrome Type 1	Autosomal Recessive
CISD2	CISD2	Wolfram Syndrome Type 2	Autosomal Recessive

## Limitation

The diagnosis of diabetes associated with mitochondrial dysfunction and endoplasmic reticulum (ER) stress is subject to several limitations. The rarity of these forms and their pronounced phenotypic heterogeneity complicate the diagnostic process and frequently lead to misclassification within the spectrum of type 1 or type 2 diabetes. Confirmatory diagnosis often requires costly genetic or molecular analyses, which are not widely available in routine laboratory settings. Existing evidence is largely derived from small case series or retrospective studies, with a notable lack of long-term, controlled prospective investigations. In clinical practice, the unpredictability of therapeutic responses further complicates management, underscoring the need for personalized treatment strategies and collaborative, multidisciplinary research efforts.
